# Extinction Risks and the Conservation of Madagascar's Reptiles

**DOI:** 10.1371/journal.pone.0100173

**Published:** 2014-08-11

**Authors:** Richard K. B. Jenkins, Marcelo F. Tognelli, Philip Bowles, Neil Cox, Jason L. Brown, Lauren Chan, Franco Andreone, Alain Andriamazava, Raphali R. Andriantsimanarilafy, Mirana Anjeriniaina, Parfait Bora, Lee D. Brady, Elisoa F. Hantalalaina, Frank Glaw, Richard A. Griffiths, Craig Hilton-Taylor, Michael Hoffmann, Vineet Katariya, Nirhy H. Rabibisoa, Jeannot Rafanomezantsoa, Domoina Rakotomalala, Hery Rakotondravony, Ny A. Rakotondrazafy, Johans Ralambonirainy, Jean-Baptiste Ramanamanjato, Herilala Randriamahazo, J. Christian Randrianantoandro, Harison H. Randrianasolo, Jasmin E. Randrianirina, Hiarinirina Randrianizahana, Achille P. Raselimanana, Andriambolantsoa Rasolohery, Fanomezana M. Ratsoavina, Christopher J. Raxworthy, Eric Robsomanitrandrasana, Finoana Rollande, Peter P. van Dijk, Anne D. Yoder, Miguel Vences

**Affiliations:** 1 Global Species Programme, IUCN, Cambridge, United Kingdom; 2 IUCN/CI Biodiversity Assessment Unit, Betty & Gordon Moore Center for Science & Oceans, Conservation International, Arlington, Virginia, United States of America; 3 IUCN Global Species Programme, Gland, Switzerland; 4 Department of Biology, Duke University, Durham, North Carolina, United States of America; 5 W. M. Keck Science Department of Claremont McKenna, Pitzer, and Scripps Colleges, Claremont, California, United States of America; 6 Museo Regionale di Scienze Naturali, Torino, Italy; 7 Ligue pour la Protection de la Nature à Madagascar, Lot 313 Cité Civil Ambohipo, Antaninarenina, Antananarivo, Madagascar; 8 Madagasikara Voakajy, Antananarivo, Madagascar; 9 WWF Madagascar and West Indian Ocean Programme Office, Antananarivo, Madagascar; 10 Département de Biologie Animale, Faculté des Sciences, Université d'Antananarivo, Antananarivo, Madagascar; 11 Calumma Ecological Services, Dunkirk, Faversham, Kent, United Kingdom; 12 Zoologische Staatssammlung München, München, Germany; 13 Durrell Institute of Conservation and Ecology, School of Anthropology and Conservation, University of Kent, Canterbury, United Kingdom; 14 IUCN Species Survival Commission, Gland, Switzerland; 15 United Nations Environment Programme World Conservation Monitoring Centre, Cambridge, United Kingdom; 16 Département de Zoologie et Ecologie, Faculté des Sciences Campus Ambondrona, Mahajanga, Madagascar; 17 Conservation International, Villa Hajanirina, Ankorahotra, Antananarivo, Madagascar; 18 Rio Tinto QMM, Fort Dauphin, Madagascar; 19 Turtle Survival Alliance, VO 12 Bis A Manakambahiny, Antananarivo, Madagascar; 20 Parc Botanique et Zoologique de Tsimbazaza, Antananarivo, Madagascar; 21 Ministère de l'Environnement et des Forêts, Nanisana, Antananarivo, Madagascar; 22 Association Vahatra, Association Vahatra, Antananarivo, Madagascar; 23 Herpetology Department, American Museum of Natural History, New York, New York, United States of America; 24 Technical University of Braunschweig, Zoological Institute, Braunschweig, Germany; Midwestern University & Arizona State University, United States of America

## Abstract

**Background:**

An understanding of the conservation status of Madagascar's endemic reptile species is needed to underpin conservation planning and priority setting in this global biodiversity hotspot, and to complement existing information on the island's mammals, birds and amphibians. We report here on the first systematic assessment of the extinction risk of endemic and native non-marine Malagasy snakes, lizards, turtles and tortoises.

**Methodology/Principal Findings:**

Species range maps from The IUCN Red List of Threatened Species were analysed to determine patterns in the distribution of threatened reptile species. These data, in addition to information on threats, were used to identify priority areas and actions for conservation. Thirty-nine percent of the data-sufficient Malagasy reptiles in our analyses are threatened with extinction. Areas in the north, west and south-east were identified as having more threatened species than expected and are therefore conservation priorities. Habitat degradation caused by wood harvesting and non-timber crops was the most pervasive threat. The direct removal of reptiles for international trade and human consumption threatened relatively few species, but were the primary threats for tortoises. Nine threatened reptile species are endemic to recently created protected areas.

**Conclusions/Significance:**

With a few alarming exceptions, the threatened endemic reptiles of Madagascar occur within the national network of protected areas, including some taxa that are only found in new protected areas. Threats to these species, however, operate inside and outside protected area boundaries. This analysis has identified priority sites for reptile conservation and completes the conservation assessment of terrestrial vertebrates in Madagascar which will facilitate conservation planning, monitoring and wise-decision making. In sharp contrast with the amphibians, there is significant reptile diversity and regional endemism in the southern and western regions of Madagascar and this study highlights the importance of these arid regions to conserving the island's biodiversity.

## Introduction

Reptiles represent a major component of vertebrate species richness in the tropics, and are the predominant group in many rainforest, arid and montane habitats [1]–[3], with a species richness pattern mainly influenced by temperature on a global scale [4]–[7]. While the threat status of other tetrapods (mammals, birds and amphibians) have been reviewed at a global scale based on full species sampling [8]–[10], reptiles (with the exception of turtles and crocodilians) have traditionally received much less attention from conservationists (but see [11]). This is particularly the case for cryptic, rare and burrowing species that are difficult to sample (e.g., [12], [13]). Many species of reptiles have small geographic ranges (e.g., [14]) and have developed special ecological adaptations and dependency on specific habitats and environmental conditions [15], [16]. These factors make reptiles highly susceptible to changes in the extent and quality of their habitats, including pollution and climate change (e.g., [17]–[21]). Exploitation of reptiles, for food, ornaments, clothing accessories and as live exhibits and pets is also a threat, especially when harvests are illegal and uncontrolled (e.g., [22]–[26]). Protected areas undoubtedly conserve important habitats for many of the world's reptiles, but certain taxa are subject to specific threats even within these protected areas as well as in areas outside of parks and reserves.

Reptiles associated with deserts, watercourses and grasslands are likely underrepresented in protected area networks that were often established primarily to conserve forests (e.g., [27]). It is therefore important to reliably identify the reptile species at greatest risk of extinction so that ameliorative actions can be designed with the aim of reducing the threats and improving their conservation status. Although efforts are underway to assess the conservation status of all the world's reptiles, analyses of the results of assessments thus far completed are limited to geographically distinct regions such as the Mediterranean Basin [28] or a randomized sample of global species [11]. Even so, the latter study in particular was successful in highlighting habitat loss and harvesting as the major global threats to reptiles, and demonstrating that threatened species are strongly associated with tropical regions [11]. However, this type of analysis, has only limited application as a conservation tool for informing regional or national conservation, because of the inevitable incomplete taxonomic coverage and it reflects a very broad-scale global pattern.

Madagascar is a priority country for conservation regardless of which criteria are used to prioritize Earth's biodiversity riches [29], [30], characterized by a rampant rate of habitat destruction [31]. There are over 370 native species of reptiles living on the island of Madagascar and its small offshore islets [32]. The vast majority of the Malagasy reptile fauna is endemic at the species level, often also at the genus level, and predominantly shares affinities with clades from Africa but also from South America [33]–[35]. Substantial effort by scientists in recent years has led to an improved understanding about taxonomy, species distributions, evolution and colonization, behaviour and population size, and the impact of habitat loss on Madagascan reptiles [32]–[33], [36]–[50], and this knowledge has been successfully used to guide conservation planning and action [51]. Conservation-related studies have mainly been inventories of the reptile fauna in protected areas [52]–[79] as summarized by D'Cruze and co-workers [42]; assessments of the relevance of reptiles for ecotourism [80]; or studies of the impact of anthropogenic influence, edge effects, climate change, and fragmentation on selected reptile communities in rainforest or dry forest [81]–[87], or of the pet trade on selected taxa [22]–[23]. Furthermore, a number of conservation-relevant studies have targeted Madagascar's chelonians [88]–[104]. However, the plethora of publications and reports has produced an important, but fragmented knowledge base, which can be awkward for conservation practioners to access and understand. In contrast to Madagascar's amphibians [44], [105]–[107], the status of the island's reptiles has so far not been comprehensively reported.

After initial overviews [108], the first major conservation assessment of the Malagasy reptiles was coordinated by Conservation International in 1995 [109], followed in 2001 by the “Conservation and Assessment Management Plan” (CAMP) workshop held at Mantasoa, Madagascar [110]. Since 2008, three major assessments of Madagascar's reptiles took place: a subset of species was assessed by taxon experts according to IUCN criteria in the framework of the study of Böhm and co-workers [11], chelonians were assessed during a workshop in Antananarivo, Madagascar, in January 2008 that produced the so-called “Vision Sokatra Gasy” Action plan for turtle conservation on the island, and all remaining species were assessed by a workshop held in Antananarivo in January 2011. These three efforts led to the assignment of 373 reptile species (representing the complete reptile fauna as of December 2011) that occur in Madagascar to the threat categories according to The IUCN Red List of Threatened Species. The aim of the present study is to compile and analyze patterns concerning the extinction risk to Malagasy reptiles, both spatially and taxonomically, using the most complete taxonomic information available.

## Materials and Methods

### Dataset, inclusions and omissions

Our master data set consisted of 393 non-marine species for potential inclusion in the study. We used information on the distribution and conservation of 367 reptile species from The IUCN Red List of Threatened Species website on 1 May 2013. Twenty six species were omitted because they were either introduced, their occurrence in Madagascar is doubtful, they lacked distribution maps, they had been recently described and not yet assessed, or because Madagascar represents a small proportion of the global range (Supporting Materials 1). Species accounts for Squamata were initially prepared by some of the authors of this study, and later refined during an expert workshop held in Antananarivo during 24–28 January 2011, whilst endemic Chelonia were assessed in a workshop in Antananarivo in January 2008.

### Spatial data

The distribution maps used in this study were compiled by IUCN based on the input from experts during the assessment of the species' risk of extinction. These maps are coarse generalizations of their distributions that include localities where the species have been recorded and suitable habitat where they may occur. Although they may over or underestimate the true area of occupancy, they represent the most accurate current depiction of species' distributions. Range maps were available for 367 reptile species that were included in the analyses. Species included in the IUCN Categories Vulnerable, Endangered, and Critically Endangered are collectively referred to as threatened species throughout this study.

### Data analyses

Data were analyzed by superimposing the species range maps over a hexagonal grid with grid cells of approximately 100 km^2^, and covering the entire island of Madagascar. A species was considered present in any given hexagon if any part of its polygon range overlapped that hexagon. We considered four major spatial patterns in the analysis:

1. Species richness, calculated by counting the number of species in each hexagon;
2. Threatened species richness, calculated by counting the number of species in the IUCN Categories Vulnerable, Endangered or Critically Endangered in each hexagon; because of the uncertainty that arises from species classed as Data Deficient, IUCN guidelines recommend that three levels of reporting are used to calculate the proportion of threatened taxa [111]. In this paper we use the mid-point, but provide the alternatives in the Supplementary Materials;
3. Unusually high or low levels of threatened species compared with their total species richness, calculated as the residual from the relationship between total richness per cell for data sufficient species (i.e., the sum of all reptiles, excluding Data Deficient species) against the total number of threatened species per cell;
4. Range-size rarity, which is an estimation of the richness of species in each hexagon weighted by the size of their geographic distribution. It was calculated as the sum of the inverse of the range-size (i.e., 1/number of cells in which a species occurs) of all species present in a hexagon. This is a continuous weighting function that assigns higher weights to species with small ranges and progressively lower weights to more widespread species, which avoids a common problem when mapping endemism, where an arbitrary region or range-size threshold is used to identify endemic species.

In order to assess the degree to which Malagasy reptiles are represented in the existing protected area system, we performed a gap analysis [112]. For that purpose, we overlaid the distribution map of each reptile species onto the layer of protected areas of Madagascar. We then calculated the percentage of each species' geographic range that is within protected areas and identified those species that are not represented within the reserve network.

During the assessment workshops, information was collected on the scope, severity, and timing of the main threats affecting each species. This information was coded against the IUCN Threatened Classification Scheme (http://www.iucnredlist.org/technical-documents/classification-schemes/threats-classification-scheme), and helped us identify the major ongoing threats affecting Malagasy reptiles, particularly those in the Critically Endangered category.

To assess the correlation among species richness of different taxonomic groups, we performed pairwise comparisons of species richness raster datasets. Pearson correlation-coefficients among each pair of data sets were calculated following the unbiased correlation method of Dutilleul [113] using the software Spatial Analysis in Macroecology [114]. This method reduces the degrees of freedom for each richness landscape according to the level of spatial autocorrelation, and thus reduces the confounding effects associated with non-independence of richness data throughout a landscape.

## Results

### Proportions of threatened species

Thirty-nine percent of the data sufficient Malagasy reptiles in our analyses are threatened with extinction (Table 1), falling in either the Critically Endangered (n = 22), Endangered (n = 49) or Vulnerable (n = 59) categories (Table S2). A further 43 species were Near Threatened and 39 were Data Deficient, whilst 155 were considered as Least Concern (Table S3).

**Table 1:** The number of Malagasy reptile species in each family assigned to the IUCN Red List categories.

Family	Critically Endangered	Endangered	Vulnerable	Near Threatened	Least Concern	Data Deficient	Total	Threatened species	Percentage threatened species
Chamaeleonidae	4	19	18	12	19	4	76	41	54
Gekkonidae	5	16	15	13	40	8	97	36	37
Scincidae	5	6	11	6	29	13	70	22	31
Gerrhosauridae	0	1	5	2	10	0	18	6	33
Opluridae	0	0	0	0	7	0	7	0	0
Psammophiidae	0	0	0	0	1	0	1	0	0
Lamprophiidae	2	7	10	10	43	5	77	19	25
Xenotyphlopidae	1	0	0	0	0	1	2	1	50
Boidae	0	0	0	0	3	0	3	0	0
Typhlopidae	0	0	0	0	3	8	11	0	0
Podocnemididae	1	0	0	0	0	0	1	1	100
Testudinidae	4	0	0	0	0	0	4	4	100

**Figure 1:**
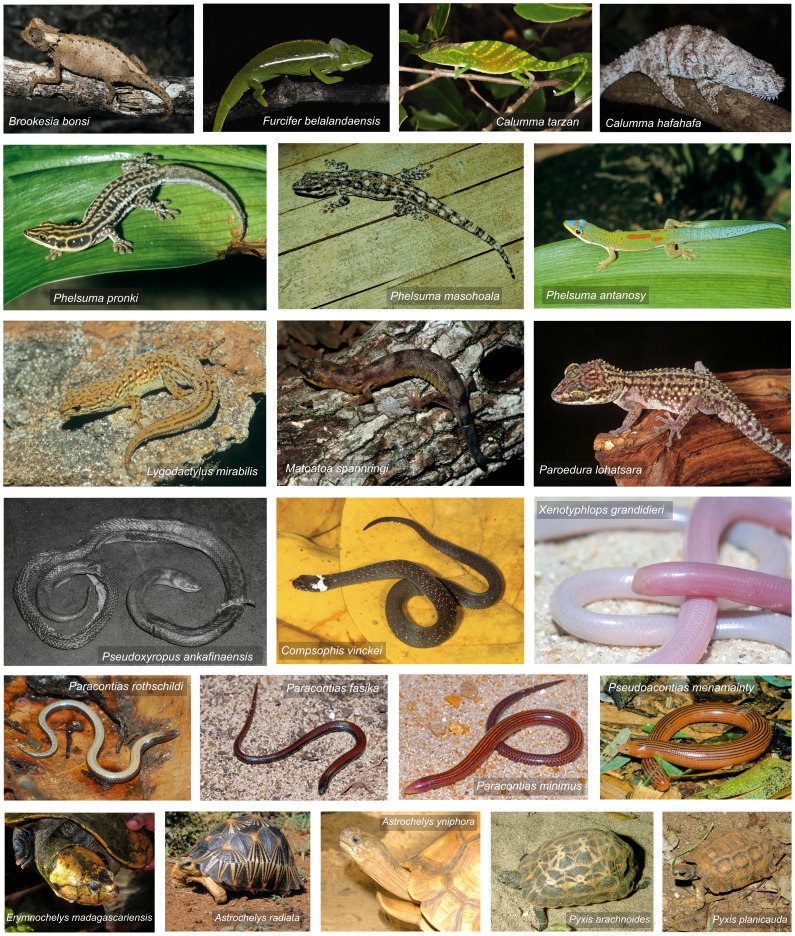
The 22 Critically Endangered species of Malagasy reptiles.

Seven families contained species listed as Critically Endangered (Table 1; Figure 1), including all species of Malagasy Testudinidae (tortoises) and Podocnemididae (turtles). Chamaeleonidae, Scincidae and Gekkonidae had the most species that are Critically Endangered, representing 5%, 7% and 5% of the respective families (Table 1). Chamaeleonidae had the highest proportion of threatened and Near Threatened species. The proportion of species listed as Data Deficient was generally low for most families (<7%), with the exception of Typhlopidae (70%) and Scincidae (18%). Taking all threat categories together (Table 1), all species in the two chelonian families Podocnemididae and Testudinidae were included in one of these categories, while this applied to only 54% of Chamaeleonidae and 31–37% of Gekkonidae, Scincidae and Gerrhosauridae. Only 25% of lamprophiid snakes and none of the boid snakes were classified as threatened. No blind snakes (Typhlopidae) were classified as threatened but this family contained a large proportion of data deficient species (8 out of 11).

### Spatial patterns

**Figure 2:**
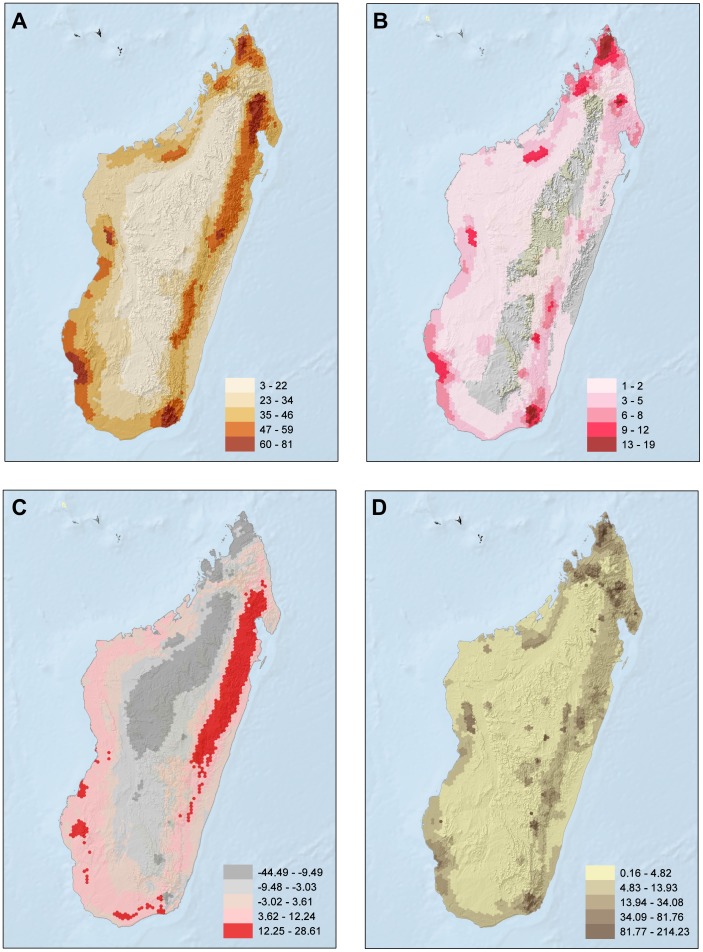
Spatial patterns for all reptile species included in this assessment. A) species richness; B) richness of threatened species; C) residuals of the relationship between threatened species and total number of species (positive values were mapped in red, indicating cells that have more threatened species than expected for their richness alone, and equal or negative values in gray, indicating cells that have the same or fewer threatened species as/than expected for richness alone); D) richness of range-size rarity.

Figure 2 presents the spatial results of this study across all taxonomic groups. Highest species richness (Figure 2a) is seen in the coastal and peripheral zones with surviving natural vegetation. Peaks occur in the far north (around Montagne d'Ambre), the north-east (between Makira and Marojejy), the central east (around Moramanga), the west (at Bemaraha), the south-west (around Ifaty) and the south-east around Tolagnaro. Species richness was lowest in the interior High Plateau, and includes all the major massif systems in Madagascar except Montagne d'Ambre, Marojejy, Anjanaharibe-Sud, and the Anosy Mountains (including Andohahela). The pattern of threatened species richness is similar to the total species richness, with the main exception being a small inland area in the south-east around Ranomafana (Figure 2b).

The residuals (unusually high or low levels of threatened species compared to total species richness) revealed a different pattern (Figure 2c). The following regions all had the highest levels of threatened species: both humid and arid areas around Montagne d'Ambre, the Sambirano region, Ankarafantsika and the Anosy Mountains. Other areas of higher than average threatened species richness included Tsaratanana, Marojey/Anjanaharibe-Sud, Masoala, Bemaraha, Ranomafana, Andringintra and the Toliara region. By contrast, the eastern humid forest, from Makira in the north to roughly the Mangoro river in the south, is the largest area identified as having a lower than average richness of threatened species. In addition, the lowland dry forests and associated habitats in the Atsimo Andrefana Region, north of Toliara; and further to the east, the coastal and inland dry forests of the Anosy and Androy Regions have a lower than average diversity of threatened species.

Finally, for range size rarity, this pattern is very similar to the threatened species richness (Figure 2d). The minor differences were mostly restricted to identifying interior regions with range size rarity, such as Isalo, Itremo Ibity and Tsaratanana and the forests linking Ankarafantsika National Park in the Boeny Region with areas to the north.

**Figure 3:**
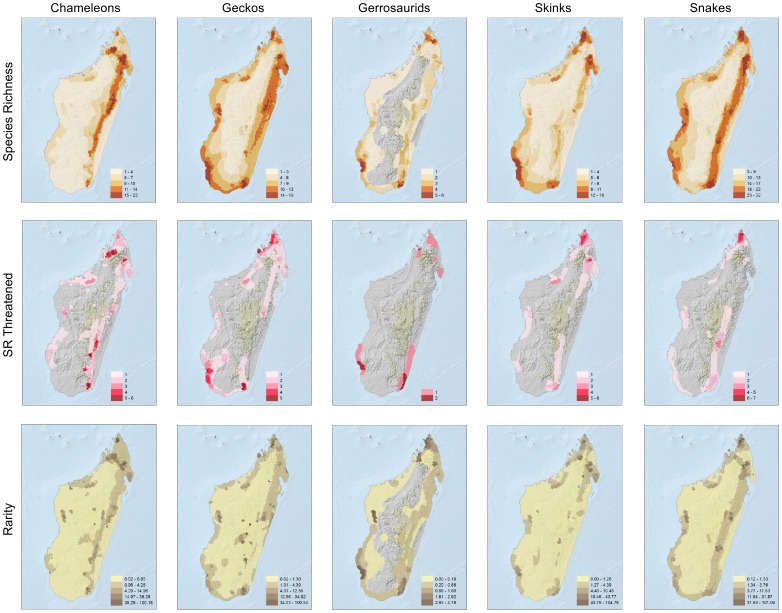
Species richness, species richness of threatened species, and range-size rarity calculated separately for five major Madagascan reptile groups. See Materials and Methods for an explanation of the metrics used.

Figure 3 presents the spatial results for each of five major reptile groups: chameleons, geckos, gerrhosaurids, skinks, and snakes. Patterns are largely similar between each group, and congruent with the total species analyses. The spatial species richness values of all the reptile groups were significantly correlated to each other (Supporting Materials Table S4), suggesting similar factors affected the diversification of each of these groups and most share areas of high and low richness. The most striking exceptions are: (i) chameleons, which have much lower levels of total and threatened species richness and range size rarity in the west, and (ii) gerrhosaurids which have low levels of total and threatened species richness, and range size rarity, in the eastern and northeastern humid forests.

### Threats to all Species and CR Species

**Figure 4:**
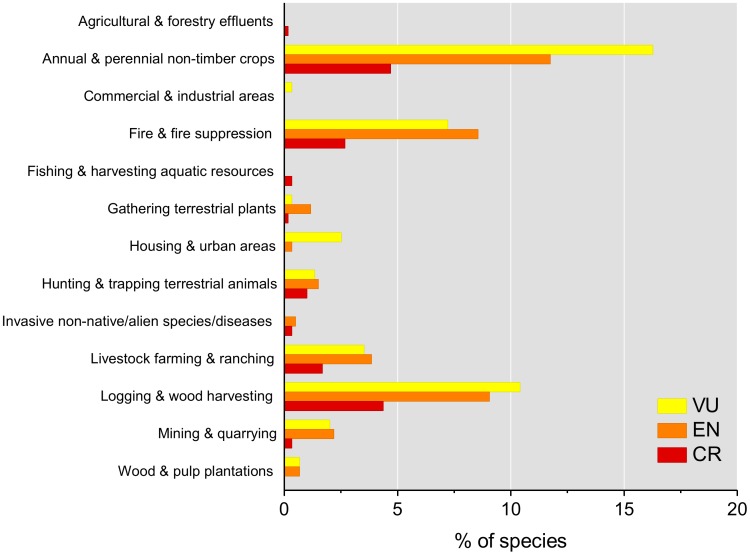
Major ongoing threats currently affecting Madagascar's reptiles.

A summary of the percentage of threatened species impacted by major ongoing threats is given in Figure 4. Habitat loss and degradation caused by expanding agriculture (annual and perennial non-timber crops), followed by logging and wood harvesting, affect the most species. Analyzing the species' accounts of the Critically Endangered reptiles in more detail, reveals that the direct removal of trees, or other plants, constitutes a threat for 55% of the 22 species in this category. Slash and burn conversion of forest and scrubland into agriculture threatens 15 (68%) of Critically Endangered species, including tortoises, snakes, skinks, chameleons and geckos, making it the most pervasive threat to reptiles in Madagascar. Fire, either directly from human set bushfires (usually to create new pasture for grazing) or as a consequence of careless honey harvesting threatens seven Critically Endangered species and is the principal threat to a montane gecko. Mineral extraction (both legal and illegal, industrial and artisanal) directly threatens five Critically Endangered species. Harvesting of wild reptiles as bushmeat for consumption in Madagascar threatens three chelonian species, whilst six other species are subject to illegal collection for the international pet trade.

### *Importance of Protected Areas*

**Figure 5:**
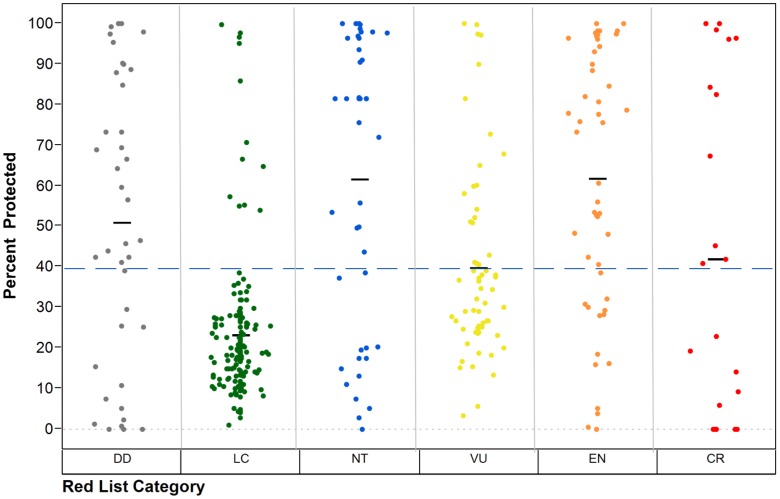
Percentage of reptile species' range represented in all Madagascar Protected Areas by IUCN Red List Category. Dotted line indicates grand mean and black short lines indicate mean percentage of range protected in each IUCN Red List Category.

**Figure 6:**
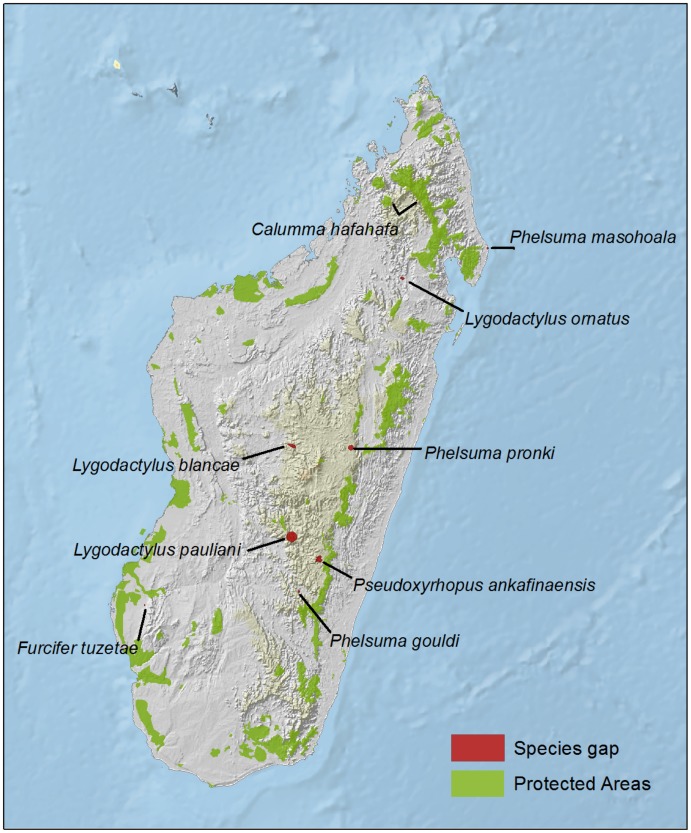
Geographic distribution of the 11 species that are not represented in any protected area (i.e., gap species).

On average, almost 40% of the geographic range of Malagasy reptiles is within some form of protected areas (Figure 5). Among threatened species, the coverage is variable, being lowest for Critically Endangered and Vulnerable species. Based on this study, only four Critically Endangered (*Calumma hafahafa*, *Phelsuma masohoala*, *P. pronki* and *Pseudoxyrhopus ankafinaensis*) and one Endangered (*Lygodactylus ornatus*) species are not represented in any formal protected area (Figure 6). However, a further nine Critically Endangered species are included within the network only through their presence in recently established, or provisional, protected areas (*C. tarzan*, *F. belalandaensis*, *L. mirabilis*, *Paracontias fasika*, *P. rothschildi*, *P. minimus*, *Xenotyphlops grandidieri*, *Phelsuma antanosy* and *Pseudoacontias menamainty*).

## Discussion

This study is the first to analyze comprehensively the conservation status of, and threats to, the endemic and native reptiles of Madagascar. It complements previous, single taxon studies and field surveys, as well as a recent assessment of a random sample of global reptile species [11]. This study highlights the major threats to Malagasy reptiles and identifies the remaining areas of native vegetation of most importance to conserving threatened reptiles, as well as demonstrating the important role that the island's new protected areas are making.As the original assessments for this study were carried out in a workshop in early 2011, new species described and taxonomic changes proposed after this date were not considered (see Table S1). In general all species of Malagasy reptiles appear to persist at the time of the assessment except one snake species (*Pseudoxyrhopus ankafinaensis*) that is possibly extinct [115]. Especially in the case of fossorial species of Scincidae, Typhlopidae and Xenotyphlopidae, our assessment relies on their apparently very restricted ranges but these animals are highly difficult to detect during fieldwork [116]–[117]. A significant range size extension has recently been published for at least one Critically Endangered species [118] but several of the newly described species not included in the assessment are likely micro-endemic to very small ranges in north-western or northernmost Madagascar, thus probably increasing the number of threatened species overall, and in particular in northern Madagascar [14], [119]–[120]. Other new or resurrected species have large ranges and are unlikely to be threatened, such as *Furcifer major* and *F. viridis* [121].

### Proportion of Threatened Species of Malagasy Reptiles

The proportion of threatened species differs among the major groups considered here, pointing to different conservation priorities. Particular attention needs to be focused on podocnemidid and testudinid chelonians as all endemic species in these taxa are threatened. Roughly half of all chameleon species are threatened which might be a combined effect of including many range-restricted ground chameleons (*Brookesia*) and in general a high dependence on forest habitat in the majority of species. Three other species-rich groups, geckos, skinks and gerrhosaurids have roughly only a third of species threatened, but geckos and skinks each contain five CR species that require particular attention. Only about a quarter of snake species are threatened (Boidae, Psammophiidae, Lamprophiidae) and only two species are CR, reflecting that non-fossorial Malagasy snakes often have wide ranges, possibly related to their body sizes that on average are larger than in lizards (M. Vences, unpublished analyses), and many species are not strictly dependent on undisturbed forest habitat. Finally, more research should be focused on blindsnakes (Typhlopidae and Xenotyphlopidae) which in their majority are data deficient, and because of their small body size and fossorial habits might in many cases be range-restricted and habitat specialists.

### Threats to Malagasy Reptiles

Twenty-two endemic Malagasy reptile species are Critically Endangered, and thus face the highest extinction risk using the IUCN categories and criteria. Broadly, the main threats are the loss of native forests, direct exploitation for food, and - mainly for tortoises - the international pet trade. With few exceptions, these are also the most prevalent threats to other Malagasy reptile species.

Of the major threat factors, habitat loss has without any doubt the most devastating effects on Madagascar's reptiles. Slash-and-burn agriculture is causing the decline or disappearance of many populations of reptiles, but it is striking that only a few studies have systematically addressed this phenomenon. Forest-specialized species comprise the vast majority of Malagasy reptiles, and their disappearance after forest destruction is so obvious that it apparently has not attracted the interest of researchers, although better understanding fragmentation effects [84]–[85] and survival of reptiles in forests of different degree of degradation, or secondary forest [87], would be of high importance.

Whilst protected areas can provide effective conservation in Madagascar it is clear that habitat loss and direct exploitation of reptiles occurs within their boundaries. Slash-and-burn and logging for timber, whilst constituting a major threat to reptiles [87], negatively impacts a range of other species, as well as ecosystem services.

In contrast to the generalized effects of habitat destruction, direct exploitation is of high relevance only for a few species in high demand as bushmeat or in the pet trade, such as chelonians, some chameleons and certain geckos. Only a few Malagasy reptile species are consumed by people for food but harvest levels are believed to be high enough to threaten extirpation of local populations. Madagascar's only endemic freshwater turtle, *Erymnochelys madagascariensis*, is subject to direct exploitation and by-catch pressure throughout its range [88], [102]. Although there is a paucity of data on population size and trends, harvest pressure appears to constitute the main threat to this species. The radiated tortoise *Astrochelys radiata* is currently mainly threatened from a massive increase of collecting large-bodied individuals for bushmeat and, to a lesser degree, for the illegal export of small-bodied tortoises for the international pet trade [95], [100]–[101]. The same is true for the spider tortoise *Pyxis arachnoides* occurring in coastal areas of south-western and southern Madagascar where habitat loss is widespread and collection occurs for the overseas pet trade [103]–[104]. For tortoise species, such as *Astrochelys yniphora*, which occurs entirely within Baly Bay National Park (from where collection is illegal) and is a protected species (prohibiting collection for food or trade), the ongoing, and seemingly increasing, illicit trade threatens to reverse decades of conservation success. Some of Madagascar's other reptile species, notably chameleons and leaf-tailed geckoes are also illegally collected for the international pet trade [122] despite collection legal collection being permitted for some taxa. Illegal trade of reptile species from Madagascar, such as chameleons and leaf-tailed geckoes, not only potentially threatens the survival of some species, but also undermines efforts to maintain a legal and sustainable trade, with benefits to local people where possible, under the auspices of the Convention of International Trade in Endangered Species of Wild Fauna and Flora (CITES). The extent to which Malagasy reptile species that are not currently listed on CITES, such as skinks, the gerrhosaurids, some geckoes and most of the snakes, are traded internationally is poorly known because quantitative information on export quantities are difficult to obtain.

### Important Regions for Malagasy Reptiles

For reptiles, our analyses reveal the conservation importance of the following regions with combinations of a high diversity of threatened species, high species richness, and local endemism: the humid and arid areas around Montagne d'Ambre, the Sambirano region, Marojey/Anjanaharibe-Sud/Tsaratanana, Masoala, Ankarafantsika, Bemaraha, Ranomafana, Andringitra, the Toliara area and the Anosy mountains. These sites include both arid and humid regions, sites with large or little topographic relief, and sites with different human impacts; which suggests that multiple biogeographic processes have contributed to these patterns of richness and endemism, and support our findings that reptiles face a broad diversity of threats.

A previous analysis of priority areas for expanding the global protected areas network included only tortoises and turtles [123] while a more targeted evaluation of priority areas for biodiversity conservation on Madagascar, across a range of taxa, only included two reptile (gecko) genera [51]. However, there is a broad congruence between the results of this study and our results, with all of our top priority reptile regions also represented in the top 10% unconstrained priority conservation areas in the previous study [51]. Other important areas identified [51] include the coastal region around Mahajanga, the north-eastern littoral forests, High Plateau massifs including Ankaratra, Ibity and Itremo, the Morondava area, and the Isalo Massif. All these regions also include endemic reptile species, and thus are important for reptile conservation. However, our study demonstrates that these sites include comparatively fewer threatened reptile species compared to our highest priority sites.

In comparison with the earlier conservation assessment of amphibians and reptiles [109], all our top priority sites were also identified in this 1995 assessment as ‘critical sites of confirmed interest’ with the single exception of Bemaraha, which was considered at this time as a ‘priority site for research’. Subsequently, there have been several herpetological surveys conducted at Bemaraha (e.g. [62], [75]). Most of the other critical sites identified by this workshop are similar to those previously included in the top 10% unconstrained priority conservation areas [51].

In comparison with the IUCN amphibian assessment of Madagascar [105], [106], all our top priority reptile conservation areas were also identified as having high diversities of threatened amphibians, with the exception of the three arid sites: Ankarafantsika, Bemaraha, and the Toliara area, which have far fewer amphibian species compared to the humid regions of Madagascar. The obvious, much lower species richness of amphibians compared to reptiles in the arid regions of Madagascar, highlights major ecological differences between these two groups, which strongly argues for them to be considered separately in conservation assessments. Other important areas for threatened amphibians [105] included the humid mid-altitude eastern rainforests, the littoral forests in the NE, and the massifs of Ankaratra, and Isalo, similar to the findings of Kremen and co-workers [51].

### Malagasy Reptiles and New Protected Areas

Madagascar has a large, and expanding, protected area network. It consists of a long-established set of national parks and other reserves managed by Madagascar National Parks, and a growing network of other protected areas managed by other entities. The reserves managed by Madagascar National Parks protect essential habitat for many endemic reptiles and have high species richness. For example, the Critically Endangered *Brookesia bonsi* is only known from Namoroka National Park in the west.

This study shows the importance of the new suite of protected areas, both in conserving overall reptile species richness and to the survival of particular Critically Endangered species. For example, *Calumma tarzan* appears to be endemic to a few small rainforest fragments in the central east [124] which are under creation as new protected areas [125].

Five fossorial Critically Endangered species (four skinks *Madascincus arenicola*, *Paracontias fasika*, *P. minimus*, and *P. rothschildi* and a blind snake *Xenotyphlops grandidieri*) are entirely, or mostly, restricted to a single area of suitable sandy habitat in extreme northern Madagascar near Antsiranana. Although this, and a nearby area (i.e. Montagne des Français, a karstic massif that harbors a large number of locally endemic and highly threatened reptile species e.g., [14], [119], [126]), are subject to ongoing work to establish their protected area status, particular, and immediate, attention should be given to conserving the micro-endemic reptiles and other threatened taxa that depend on these small, unique ecosystems [119].

Other areas specifically important for micro-endemic threatened reptile species are the summits of the Ankaratra and Ibity massifs (for the geckos *Lygodactylus mirabilis* and *L. blanci*, respectively). Recent descriptions of one chameleon (*Brookesia brunoi*) and one gecko (*Phelsuma gouldi*), both not yet included in the present assessment, also highlight the importance of the last remaining fragments of forest on the southern central high plateau, especially in the private Anja Reserve, near Ambalavao [127]–[128]. Sites of major importance are those that hold the entire known population of a threatened reptile species. Some of these, such as the ploughshare tortoise *Astrochelys yniphora* from Baly Bay National Park, are already included on the Alliance of Zero Extinction (AZE) database [128] but additional scrutiny is needed to determine other AZE sites with the results of this study.

### The Next Decade 2014–2024

This study, and the results of Böhm and co-workers' [51], provide a strong basis for progressing reptile conservation, at local, national and international levels. Based on the results of our analysis, we tentatively put forward a series of priority actions for the conservation of Malagasy reptiles. This is not meant to be an exhaustive list, but any progress made on these actions will deliver specific conservation benefits to the endemic reptiles of Madagascar.

As a priority action, conservation effort should be directed to those areas standing out as particular conservation hotspots for reptiles in Madagascar. This includes efforts to reduce deforestation and habitat degradation through strengthened law enforcement and building the capacity of local communities to pursue sustainable livelihood opportunities in and around existing protected areas.

Furthermore, strategies should be developed to conserve the habitats of those threatened species occurring in the yet largely unprotected areas such as the dunes around Antsiranana, the Ankaratra Massif and the forests known to harbour populations of *Calumma tarzan* and *Matoatoa spannringi*.

We also identify three scientific research priorities related to the conservation of Malagasy reptiles:

1. Continued exploration of taxonomy and diversity of reptile species. A large number of candidate species of reptiles have already been identified [129], and many other nominal species are deeply subdivided genetically (e.g., [129]–[132]) or otherwise require taxonomic revision [129]. It is important to continue this work to gain a realistic view of Madagascar's reptile diversity and, consequently, its threat status;
2. Assess the vulnerability of Malagasy reptiles to climate change. It is important to determine whether the reported upward elevational shift of Madagascar's montane reptiles, associated with regional climate warming [86], is a general pattern in Madagascar's massifs, and at what time scale such climate change-driven factors might constitute an extinction threat [133];
3. Remedy the surprising lack of studies on the effects of logging and forest degradation on reptiles, and on their diversity in degraded and secondary habitats of Madagascar.;

We have highlighted the need for effective *in situ* conservation and the priority gaps to be addressed by scientific research. Finally, we also propose four actions targeted at collaboration and stakeholder engagement:

1. Initiate, and sustain, a Government-led campaign to reduce the illegal harvest of tortoises for the domestic bush meat trade;
2. Improve international cooperation and law enforcement to significantly reduce the illegal trade of tortoises and lizards from Madagascar;
3. Improve collaboration between local communities, scientists and Government to support non-detrimental, legal and equitable international trade in certain reptile species;
4. Herpetologists to communicate the importance of certain sites for highly threatened reptiles to the Malagasy government, other zoologists and botanists and stakeholders engaged in, or dependent on, the conservation of the site. This is likely to be particularly important in some of the new or provisional protected areas

## Supporting Information

Table S1 **Reptile species distributed in Madagascar, or thought to occur in Madagascar, that were omitted from the analyses.**
(DOCX)

Table S2 **The status of globally threatened Malagasy reptiles accessed from The IUCN Red List of Threatened Species on 1 May 2013.**
(DOCX)

Table S3 **Malagasy reptile species classified as Near Threatened, Least Concern, and Data Deficient.**
(DOCX)

Table S4 **Correlations among species richness in major taxonomic reptile groups.**
(DOCX)

Text S1 **Comments on taxonomy and specific threats to selected species.**
(DOCX)
