# Supplementary material for: Extinction Risks and the Conservation of Madagascar's Reptiles
Source: PLoS One. 2014 Aug 11;9(8):e100173. doi: 10.1371/journal.pone.0100173 (PMC4128600; doi:10.1371/journal.pone.0100173)
Supplement: Table S1 — Reptile species distributed in Madagascar, or thought to occur in Madagascar, that were omitted from the analyses. (DOCX) [file pone.0100173.s001.docx]

**Richard K. B. Jenkins et al.: Extinction Risk and Conservation of Madagascar’s Reptiles**

**Supporting Materials – Table S1.** Non-marine reptile species known, or thought, to occur in Madagascar that were omitted from the analyses.

| **Family** | **Genus** | **Species** | **Red List Category** | **Status** | **Reason for omissions** |
| --- | --- | --- | --- | --- | --- |
| AGAMIDAE | *Agama* | *agama* | LC | Introduced | Occurrence doubtful in Madagascar |
| BOIDAE | *Sanzinia* | *volontany* | NE | Endemic | Recent elevation to species level |
| CROCODYLIDAE | *Crocodylus* | *niloticus* | LR/LC | Native | No maps and Madagascar represents a small proportion of the global range |
| CHAMAELEONIDAE | *Brookesia* | *brunoi* | NE | Endemic | Recent description, not yet assessed |
| CHAMAELEONIDAE | *Brookesia* | *confidens* | NE | Endemic | Recent description, not yet assessed |
| CHAMAELEONIDAE | *Brookesia* | *desperata* | NE | Endemic | Recent description, not yet assessed |
| CHAMAELEONIDAE | *Brookesia* | *micra* | NE | Endemic | Recent description, not yet assessed |
| CHAMAELEONIDAE | *Brookesia* | *tristis* | NE | Endemic | Recent description, not yet assessed |
| CHAMAELEONIDAE | *Furcifer* | *major* | NE | Endemic | Recent resurrection from synonymy, not yet assessed |
| CHAMAELEONIDAE | *Furcifer* | *viridis* | NE | Endemic | Recent description, not yet assessed |
| GEKKONIDAE | *Gehyra* | *mutilata* | NE | Introduced | Introduced species |
| GEKKONIDAE | *Hemidactylus* | *platycephalus* | LC | Introduced | Introduced species |
| GEKKONIDAE | *Hemidactylus* | *frenatus* | LC | Introduced | Introduced species |
| GEKKONIDAE | *Matoatoa* | *spannringi* | CR | Endemic | Requires reassessment |
| GEKKONIDAE | *Phelsuma* | *dubia* | NE | Native | No account and no map available |
| GEKKONIDAE | *Phelsuma* | *cepediana* | LC | Introduced | Occurrence doubtful in Madagascar |
| GERRHOSAURIDAE | *Zonosaurus* | *maramaintso* | DD | Endemic | No locality known and no map available |
| LAMPROPHIIDAE | *Liopholidophis* | *baderi* | NE | Endemic | Recent description, not yet assessed |
| LAMPROPHIIDAE | *Liopholidophis* | *oligolepis* | NE | Endemic | Recent description, not yet assessed |
| LAMPROPHIIDAE | *Madagascarophis* | *fuchsi* | NE | Endemic | Recent description, not yet assessed |
| PELOMEDUSIDAE | *Pelomedusa* | *subrufa* | LC | Introduced? | IUCN species account in draft until completed for remainder of its global range |
| PELOMEDUSIDAE | *Pelusios* | *castanoides* | LC | Globally/regionally widespread native | Might be introduced and main range is outside of Madagascar |
| PELOMEDUSIDAE | *Pelusios* | *subniger* | LC | Globally/regionally widespread native | Might be introduced and main range is outside of Madagascar |
| SCINCIDAE | *Cryptoblepharus* | *boutonii* | LC | Native | Serious taxonomic problems exist with this species; account still in draft assuming a widespread non-endemic |
| SCINCIDAE | *Pseudoacontias* | *madagascariensis* | DD | Endemic | No locality known and no map available. |
| SCINCIDAE | *Sirenoscincus* | *mobydick* | NE | Endemic | Recent description, not yet assessed |
| SCINCIDAE | *Madascincus* | *intermedius* | DD | Synonym of *Madascincus polleni* | This species was assessed by the Sampled Red List Index so is on the published Red List as DD. It was reassessed at the workshop as LC. It is no longer considered a valid taxon. |
| SCINCIDAE | *Trachylepis* | *betsileana* | DD |  | Dubious species, occurrence in Madagascar doubtful |
| SCINCIDAE | *Trachylepis* | *comorensis* | LC | Introduced | Introduced species |
| TESTUDINIDAE | *Kinixys* | *zombensis* | NE | Introduced | Introduced species |
| TYPHLOPIDAE | *Ramphotyphlops* | *braminus* | LC | Introduced | Introduced species |
