# Supplementary material for: Extinction Risks and the Conservation of Madagascar's Reptiles
Source: PLoS One. 2014 Aug 11;9(8):e100173. doi: 10.1371/journal.pone.0100173 (PMC4128600; doi:10.1371/journal.pone.0100173)
Supplement: Table S2 — The status of globally threatened Malagasy reptiles accessed from The IUCN Red List of Threatened Species on 1 May 2013. (DOCX) [file pone.0100173.s002.docx]

**Richard K. B. Jenkins et al.: Extinction Risk and Conservation of Madagascar’s Reptiles**

**Supporting Material – Table S2.** The status of globally threatened Malagasy reptiles accessed from The IUCN Red List of Threatened Species on 1 May 2013. Data on trade are based on our own, cursorial surveys of specimens offered in the pet trade especially in Europe but also in the USA over the past 10-15 years; these data are therefore not quantitative and might be incomplete, but overall should give a good representation of the trade also of non-CITES species (which often are incorrectly declared on official export permits due to changing taxonomy and difficult identification). Trade: n = no official exports in recent years; y = yes (official exports); ILL = illegal exports in recent years; HD = potentially high demand; BIC = international breeding stocks in captivity. It is illegal to collect species classed as Strictly Protected under Malagasy law (SP below) whilst other species, (MP below) can be collected and traded with permission from the government of Madagascar.

| **Species** | **Family** | **Red-list criterion** | **Occurrence in pet trade** | **CITES listing** | **Malagasy Legislation** |
| --- | --- | --- | --- | --- | --- |
| **Critically Endangered** |  |  |  |  |  |
| *Brookesia bonsi* | Chamaeleonidae | N/A | n | II | MP |
| *Calumma hafahafa* | Chamaeleonidae | B1ab(iii) | n | II |  |
| *Calumma tarzan* | Chamaeleonidae | B1ab(iii)+2ab(iii) | n, HD? | II |  |
| *Furcifer belalandaensis* | Chamaeleonidae | N/A | n | II | MP |
| *Lygodactylus mirabilis* | Gekkonidae | B1ab(iii) | n?, BIC? | -- |  |
| *Paroedura lohatsara* | Gekkonidae | B1ab(iii) | n, BIC | -- |  |
| *Phelsuma antanosy* | Gekkonidae | B1ab(ii,iii)+2ab(ii,iii) | n, HD | II | MP |
| *Phelsuma masohoala* | Gekkonidae | B1ab(iii)+2ab(iii) | n | II | MP |
| *Phelsuma pronki* | Gekkonidae | B1ab(iii) | n, BIC?, HD | II | MP |
| *Compsophis vinckei* | Lamprophiidae | B1ab(iii) | n | -- |  |
| *Pseudoxyrhopus ankafinaensis* | Lamprophiidae | B1ab(iii) | n | -- |  |
| *Erymnochelys madagascariensis* | Podocnemididae | A4d | n, HD | II | MP |
| *Madascincus arenicola* | Scincidae | B1ab(iii) | n |  |  |
| *Paracontias fasika* | Scincidae | B1ab(iii) | n | -- |  |
| *Paracontias minimus* | Scincidae | B1ab(iii) | n | -- |  |
| *Paracontias rothschildi* | Scincidae | B1ab(iii) | n | -- |  |
| *Pseudoacontias menamainty* | Scincidae | B1ab(iii) | n | -- |  |
| *Astrochelys radiata* | Testudinidae | A4d, E | ill, HD, BIC | I | SP |
| *Astrochelys yniphora* | Testudinidae | A4ad; B2ab(v); C1; E | ill, HD | I | SP |
| *Pyxis arachnoides* | Testudinidae | A4cd; E | ill, HD, BIC | I | SP |
| *Pyxis planicauda* | Testudinidae | A4acd | ill, HD, BIC | I | SP |
| *Xenotyphlops grandidieri* | Xenotyphlopidae | B1ab(iii)+2ab(iii) | n | -- |  |
| **Endangered** |  |  |  |  |  |
| *Brookesia bekolosy* | Chamaeleonidae | N/A | n | II | MP |
| *Brookesia decaryi* | Chamaeleonidae | B1ab(iii) | n | II | MP |
| *Brookesia dentata* | Chamaeleonidae | B1ab(iii) | n | II | MP |
| *Brookesia exarmata* | Chamaeleonidae | N/A | n | II | MP |
| *Brookesia karchei* | Chamaeleonidae | N/A | n | II | MP |
| *Brookesia lineata* | Chamaeleonidae | N/A | n | II | MP |
| *Brookesia perarmata* | Chamaeleonidae | B1ab(iii) | n, HD | I | SP |
| *Brookesia ramanantsoai* | Chamaeleonidae | N/A | n | II | MP |
| *Brookesia valerieae* | Chamaeleonidae | N/A | n | II | MP |
| *Calumma andringitraense* | Chamaeleonidae | N/A | n | II |  |
| *Calumma furcifer* | Chamaeleonidae | B1ab(iii) | n | II |  |
| *Calumma gallus* | Chamaeleonidae | N/A | n, HD | II | MP |
| *Calumma glawi* | Chamaeleonidae | B1ab(iii) | n | II | MP |
| *Calumma globifer* | Chamaeleonidae | N/A | n | II | MP |
| *Calumma hilleniusi* | Chamaeleonidae | D2 | n | II | MP |
| *Calumma vencesi* | Chamaeleonidae | N/A | n | II |  |
| *Furcifer balteatus* | Chamaeleonidae | B1ab(iii,v) | n | II | MP |
| *Furcifer minor* | Chamaeleonidae | B1ab(iii) | n, HD | II | MP |
| *Furcifer nicosiai* | Chamaeleonidae | N/A | n | II | MP |
| *Ebenavia maintimainty* | Gekkonidae | N/A | n | -- |  |
| *Lygodactylus intermedius* | Gekkonidae | B1ab(iii,v)+2ab(iii,v) | n | -- |  |
| *Lygodactylus ornatus* | Gekkonidae | B1ab(iii) | n | -- |  |
| *Lygodactylus roavolana* | Gekkonidae | B1ab(iii) | n | -- |  |
| *Paragehyra gabriellae* | Gekkonidae | B1ab(iii) | n | -- |  |
| *Paroedura masobe* | Gekkonidae | B1ab(iii) | ??, BIC?, HD | -- |  |
| *Paroedura tanjaka* | Gekkonidae | B1ab(iii) | n | -- |  |
| *Phelsuma flavigularis* | Gekkonidae | B1ab(iii) | n | II | MP |
| *Phelsuma klemmeri* | Gekkonidae | B1ab(iii) | n, BIC | II |  |
| *Phelsuma roesleri* | Gekkonidae | B1ab(iii) | n, HD | II |  |
| *Phelsuma seippi* | Gekkonidae | B1ab(iii) | n, BIC | II | MP |
| *Phelsuma serraticauda* | Gekkonidae | B1ab(v) | n, BIC? | II | MP |
| *Phelsuma vanheygeni* | Gekkonidae | B1ab(iii) | n, BIC | II |  |
| *Uroplatus guentheri* | Gekkonidae | B1ab(iii) | ??, BIC? | II | MP |
| *Uroplatus malahelo* | Gekkonidae | B1ab(iii,v) | n | II | MP |
| *Uroplatus pietschmanni* | Gekkonidae | B1ab(iii) | ??, BIC? | II | MP |
| *Zonosaurus subunicolor* | Gerrhosauridae | B1ab(iii) | n | -- |  |
| *Alluaudina mocquardi* | Lamprophiidae | B1ab(iii) | n | -- |  |
| *Heteroliodon fohy* | Lamprophiidae | B1ab(iii) | N | -- |  |
| *Lycodryas guentheri* | Lamprophiidae | B1ab(ii,iii) | n | -- |  |
| *Lycodryas inopinae* | Lamprophiidae | B1ab(i,iii) | n | -- |  |
| *Phisalixella variabilis* | Lamprophiidae | B1ab(iii) | n | -- |  |
| *Pseudoxyrhopus kely* | Lamprophiidae | B1ab(ii,iii)+2ab(ii,iii) | n | -- |  |
| *Thamnosophis martae* | Lamprophiidae | B1ab(iii)+2ab(iii) | n | -- |  |
| *Amphiglossus decaryi* | Scincidae | B1ab(iii,v) | n | -- |  |
| *Madascincus macrolepis* | Scincidae | B1ab(iii) | n | -- |  |
| *Pseudoacontias angelorum* | Scincidae | B1ab(iii) | n | -- |  |
| *Pygomeles petteri* | Scincidae | B1ab(iii) | n | -- |  |
| *Sirenoscincus yamagishii* | Scincidae | B1ab(iii) | n | -- |  |
| *Voeltzkowia mira* | Scincidae | B1ab(iii) | n | -- |  |
| **Vulnerable** |  |  |  |  |  |
| *Brookesia ebenaui* | Chamaeleonidae | B1ab(iii) | n | II | MP |
| *Brookesia minima* | Chamaeleonidae | B1ab(iii) | n | II | MP |
| *Brookesia nasus* | Chamaeleonidae | B1ab(iii) | n | II | MP |
| *Brookesia peyrierasi* | Chamaeleonidae | B1ab(iii) | n | II | MP |
| *Brookesia tuberculata* | Chamaeleonidae | B1ab(iii) | n | II |  |
| *Brookesia vadoni* | Chamaeleonidae | B1ab(iii) | n | II | MP |
| *Calumma capuroni* | Chamaeleonidae | N/A | n | II | MP |
| *Calumma cucullatum* | Chamaeleonidae | N/A | n | II | MP |
| *Calumma jejy* | Chamaeleonidae | N/A | n | II |  |
| *Calumma oshaughnessyi* | Chamaeleonidae | N/A | n | II | MP |
| *Calumma peyrierasi* | Chamaeleonidae | B1ab(iii)+2ab(iii) | n | II | MP |
| *Calumma tsaratananense* | Chamaeleonidae | B1ab(iii) | n | II | MP |
| *Calumma tsycorne* | Chamaeleonidae | N/A | n | II |  |
| *Furcifer antimena* | Chamaeleonidae | B1ab(iii) | n | II | MP |
| *Furcifer campani* | Chamaeleonidae | B1ab(iii) | n, BIC? | II | MP |
| *Furcifer labordi* | Chamaeleonidae | N/A | n | II | MP |
| *Furcifer petteri* | Chamaeleonidae | B1ab(iii) | n | II | MP |
| *Furcifer rhinoceratus* | Chamaeleonidae | N/A | n | II | MP |
| *Blaesodactylus boivini* | Gekkonidae | N/A | y | -- |  |
| *Lygodactylus bivittis* | Gekkonidae | B1ab(iii) | n | -- |  |
| *Lygodactylus blanci* | Gekkonidae | D2 | n | -- |  |
| *Lygodactylus madagascariensis* | Gekkonidae | B1ab(iii) | n | -- |  |
| *Matoatoa brevipes* | Gekkonidae | B1ab(iii) | n | -- |  |
| *Paragehyra petiti* | Gekkonidae | D2 | n | -- |  |
| *Paroedura androyensis* | Gekkonidae | B1ab(iii) | BIC | -- |  |
| *Paroedura vazimba* | Gekkonidae | B1ab(iii) | BIC | -- |  |
| *Phelsuma breviceps* | Gekkonidae | B1ab(iii) | n | -- | MP |
| *Phelsuma hielscheri* | Gekkonidae | B1ab(iii) | BIC | II | MP |
| *Phelsuma standingi* | Gekkonidae | B1ab(iii,v) | BIC | II | MP |
| *Uroplatus ebenaui* | Gekkonidae | B1ab(iii) | y? | II |  |
| *Uroplatus giganteus* | Gekkonidae | B1ab(iii) | n? | II |  |
| *Uroplatus henkeli* | Gekkonidae | B1ab(iii) | BIC | II | MP |
| *Uroplatus malama* | Gekkonidae | B1ab(iii) | n | II | MP |
| *Tracheloptychus petersi* | Gerrhosauridae | B1ab(iii) | y | -- |  |
| *Zonosaurus anelanelany* | Gerrhosauridae | B1ab(iii) | n | -- |  |
| *Zonosaurus boettgeri* | Gerrhosauridae | B1ab(iii) | n | -- |  |
| *Zonosaurus maximus* | Gerrhosauridae | B1ab(iii,v) | n | -- |  |
| *Zonosaurus quadrilineatus* | Gerrhosauridae | B1ab(iii) | y | -- |  |
| *Brygophis coulangesi* | Lamprophiidae | B1ab(ii,iii) | n |  |  |
| *Compsophis zeny* | Lamprophiidae | B1ab(iii) | n | -- |  |
| *Liophidium therezieni* | Lamprophiidae | B1ab(iii) | n | -- |  |
| *Liopholidophis grandidieri* | Lamprophiidae | B1ab(iii) | n | -- |  |
| *Lycodryas citrinus* | Lamprophiidae | B1ab(iii,v) | n | -- |  |
| *Lycodryas inornatus* | Lamprophiidae | B1ab(iii) | n | -- |  |
| *Pararhadinaea melanogaster* | Lamprophiidae | B1ab(iii) | n | -- |  |
| *Pseudoxyrhopus oblectator* | Lamprophiidae | B1ab(iii) | n | -- |  |
| *Pseudoxyrhopus sokosoko* | Lamprophiidae | B1ab(iii) | n | -- |  |
| *Thamnosophis stumpffi* | Lamprophiidae | B1ab(iii) | n | -- |  |
| *Amphiglossus alluaudi* | Scincidae | B1ab(iii) | n | -- |  |
| *Amphiglossus anosyensis* | Scincidae | B1ab(iii) | n | -- |  |
| *Amphiglossus ardouini* | Scincidae | B1ab(iii) | n | -- |  |
| *Amphiglossus mandokava* | Scincidae | B1ab(ii,iii) | n | -- |  |
| *Amphiglossus splendidus* | Scincidae | B1ab(iii) | n | -- |  |
| *Madascincus nanus* | Scincidae | B1ab(iii) | n | -- |  |
| *Paracontias kankana* | Scincidae | B1ab(iii) | n | -- |  |
| *Pseudoacontias unicolor* | Scincidae | D2 | n | -- |  |
| *Trachylepis dumasi* | Scincidae | B1ab(iii) | n | -- |  |
| *Trachylepis lavarambo* | Scincidae | D2 | n | -- |  |
| *Trachylepis tavaratra* | Scincidae | B1ab(iii) | n | -- |  |
