# Supplementary material for: Extinction Risks and the Conservation of Madagascar's Reptiles
Source: PLoS One. 2014 Aug 11;9(8):e100173. doi: 10.1371/journal.pone.0100173 (PMC4128600; doi:10.1371/journal.pone.0100173)
Supplement: Table S3 — Malagasy reptile species classified as Near Threatened, Least Concern, and Data Deficient. (DOCX) [file pone.0100173.s003.docx]

**Richard K. B. Jenkins et al.: Extinction Risk and Conservation of Madagascar’s Reptiles**

**Supporting Materials – Table S3.** Malagasy reptile species classified as Near Threatened, Least Concern, and Data Deficient in a data set downloaded from The IUCN Red List of Threatened Species ([www.iucnredlist.org](http://www.iucnredlist.org)) on 1.5.13.

| **Family** | **Near Threatened** | **Least Concern** | **Data Deficient** |
| --- | --- | --- | --- |
| Boidae | *---* | *Acrantophis dumerili, Acrantophis madagascariensis, Sanzinia madagascariensis* | *---* |
| Chamaeleonidae | *Brookesia ambreensis, Brookesia antakarana, Brookesia betschi, Brookesia griveaudi, Brookesia lolontany, Calumma amber, Calumma ambreense, Calumma guibei, Calumma marojezense, Calumma parsonii, Calumma peltierorum, Furcifer timoni* | *Brookesia brygooi, Brookesia stumpffi, Brookesia superciliaris, Brookesia therezieni, Brookesia thieli, Calumma boettgeri, Calumma brevicorne, Calumma crypticum, Calumma gastrotaenia, Calumma guillaumeti, Calumma malthe, Calumma nasutum, Furcifer angeli, Furcifer bifidus, Furcifer lateralis, Furcifer oustaleti, Furcifer pardalis, Furcifer verrucosus, Furcifer willsii* | *Brookesia lambertoni, Calumma fallax, Calumma vatosoa, Furcifer tuzetae* |
| Gekkonidae | *Lygodactylus blancae, Lygodactylus expectatus, Lygodactylus guibei, Lygodactylus klemmeri, Lygodactylus rarus, Paroedura ibityensis, Paroedura maingoka, Paroedura oviceps, Phelsuma berghofi, Phelsuma dorsivittata, Phelsuma malamakibo, Uroplatus alluaudi, Uroplatus finiavana* | *Blaesodactylus antongilensis, Blaesodactylus sakalava, Ebenavia inunguis, Geckolepis maculata, Geckolepis typica, Hemidactylus mercatorius, Lygodactylus arnoulti, Lygodactylus heterurus, Lygodactylus miops, Lygodactylus montanus, Lygodactylus pictus, Lygodactylus tolampyae, Lygodactylus tuberosus, Lygodactylus verticillatus, Paroedura bastardi, Paroedura gracilis, Paroedura homalorhina, Paroedura karstophila, Paroedura picta, Paroedura stumpffi, Paroedura vahiny, Phelsuma abbotti, Phelsuma barbouri, Phelsuma grandis, Phelsuma guttata, Phelsuma kochi, Phelsuma laticauda, Phelsuma lineata, Phelsuma madagascariensis, Phelsuma modesta, Phelsuma mutabilis, Phelsuma parva, Phelsuma pusilla, Phelsuma quadriocellata, Phelsuma ravenala, Uroplatus fimbriatus, Uroplatus lineatus, Uroplatus phantasticus, Uroplatus sameiti, Uroplatus sikorae* | *Blaesodactylus ambonihazo, Geckolepis polylepis, Lygodactylus decaryi, Lygodactylus pauliani, Phelsuma borai,* *Phelsuma gouldi, Phelsuma hoeschi, Phelsuma kely* |
| Gerrhosauridae | *Zonosaurus haraldmeieri, Zonosaurus rufipes* | *Tracheloptychus madagascariensis, Zonosaurus aeneus, Zonosaurus bemaraha, Zonosaurus brygooi, Zonosaurus karsteni, Zonosaurus laticaudatus, Zonosaurus madagascariensis, Zonosaurus ornatus, Zonosaurus trilineatus, Zonosaurus tsingy* | *Zonosaurus maramaintso* |
| Lamprophiidae | *Compsophis albiventris, Compsophis fatsibe, Heteroliodon lava, Liophidium pattoni, Liopholidophis dimorphus, Lycodryas carleti, Pseudoxyrhopus ambreensis, Pseudoxyrhopus analabe, Pseudoxyrhopus imerinae, Thamnosophis mavotenda* | *Alluaudina bellyi, Compsophis boulengeri, Compsophis infralineata, Compsophis laphystius, Dromicodryas bernieri, Dromicodryas quadrilineatus, Exallodontophis albignaci, Heteroliodon occipitalis, Ithycyphus goudoti, Ithycyphus miniatus, Ithycyphus oursi, Ithycyphus perineti, Langaha alluaudi, Langaha madagascariensis, Langaha pseudoalluaudi, Leioheterodon geayi, Leioheterodon madagascariensis, Leioheterodon modestus, Liophidium chabaudi, Liophidium rhodogaster, Liophidium torquatum, Liophidium vaillanti, Liopholidophis dolicocercus, Liopholidophis rhadinaea, Liopholidophis sexlineatus, Liopholidophis varius, Lycodryas gaimardi, Lycodryas granuliceps, Lycodryas pseudogranuliceps, Madagascarophis colubrinus, Madagascarophis meridionalis, Madagascarophis ocellatus, Micropisthodon ochraceus, Parastenophis betsileanus, Phisalixella arctifasciata, Phisalixella tulearensis, Pseudoxyrhopus heterurus, Pseudoxyrhopus microps, Pseudoxyrhopus quinquelineatus, Pseudoxyrhopus tritaeniatus, Thamnosophis epistibes, Thamnosophis infrasignatus, Thamnosophis lateralis* | *Ithycyphus blanci, Liophidium apperti, Liophidium maintikibo, Liophidium trilineatum, Phisalixella iarakaensis* |
| Opluridae | *---* | *Chalarodon madagascariensis, Oplurus cuvieri, Oplurus cyclurus, Oplurus fierinensis, Oplurus grandidieri, Oplurus quadrimaculatus, Oplurus saxicola* | *---* |
| Psammophiidae | *---* | *Mimophis mahfalensis* | *---* |
| Scincidae | *Madascincus stumpffi, Paracontias brocchii, Paracontias hafa, Trachylepis nancycoutuae, Trachylepis volamenaloha, Voeltzkowia petiti* | *Amphiglossus astrolabi, Amphiglossus crenni, Amphiglossus frontoparietalis, Amphiglossus macrocercus, Amphiglossus melanurus, Amphiglossus ornaticeps, Amphiglossus punctatus, Amphiglossus reticulatus, Amphiglossus tanysoma, Amphiglossus tsaratananensis, Androngo trivittatus, Madascincus igneocaudatus, Madascincus melanopleura, Madascincus minutus, Madascincus mouroundavae, Madascincus polleni, Paracontias hildebrandti, Paracontias holomelas, Pygomeles braconnieri, Trachylepis aureopunctata, Trachylepis boettgeri, Trachylepis elegans, Trachylepis gravenhorstii, Trachylepis madagascariensis, Trachylepis tandrefana, Trachylepis vato, Voeltzkowia fierinensis, Voeltzkowia lineata, Voeltzkowia rubrocaudata* | *Amphiglossus andranovahensis, Amphiglossus elongatus, Amphiglossus gastrostictus, Amphiglossus mandady, Amphiglossus spilostichus, Amphiglossus stylus, Madascincus ankodabensis, Paracontias manify, Paracontias milloti, Paracontias tsararano, Paracontias vermisaurus, Pseudoacontias madagascariensis, Trachylepis vezo* |
| Typhlopidae | *---* | *Typhlops arenarius, Typhlops boettgeri, Typhlops decorsei* | *Typhlops andasibensis, Typhlops domerguei, Typhlops madagascariensis, Typhlops microcephalus, Typhlops mucronatus, Typhlops ocularis, Typhlops rajeryi, Typhlops reuteri* |
| Xenotyphlopidae | *---* | *---* | *Xenotyphlops mocquardi* |
