# Supplementary material for: Extinction Risks and the Conservation of Madagascar's Reptiles
Source: PLoS One. 2014 Aug 11;9(8):e100173. doi: 10.1371/journal.pone.0100173 (PMC4128600; doi:10.1371/journal.pone.0100173)
Supplement: Table S4 — Correlations among species richness in major taxonomic reptile groups. (DOCX) [file pone.0100173.s004.docx]

**Richard K. B. Jenkins et al.: Extinction Risk and Conservation of Madagascar’s Reptiles**

**Supporting Materials – Table S4.** Correlations among species richness in major taxonomic reptile groups, calculated after reducing spatial autocorrelation (Dutilleul 1993). Significant correlations are marked in italics.

| **Comparison** |  | **R value** | **Corrected P-value** |
| --- | --- | --- | --- |
| chameleons | geckos | 0.502 | *<0.001* |
| geckos | gerrhosaurids | 0.733 | *<0.001* |
| geckos | skinks | 0.717 | *<0.001* |
| geckos | snakes | 0.805 | *<0.001* |
| chameleons | gerrhosaurids | 0.457 | *<0.001* |
| chameleons | skinks | 0.457 | *<0.001* |
| chameleons | snakes | 0.602 | *<0.001* |
| skinks | snakes | 0.71 | *<0.001* |
| gerrhosaurids | skinks | 0.678 | *<0.001* |
| gerrhosaurids | snakes | 0.78 | *<0.001* |
| all rarity | all endemicty | 0.549 | *<0.001* |
