# Supplementary material for: Extinction Risks and the Conservation of Madagascar's Reptiles
Source: PLoS One. 2014 Aug 11;9(8):e100173. doi: 10.1371/journal.pone.0100173 (PMC4128600; doi:10.1371/journal.pone.0100173)
Supplement: Text S1 — Comments on taxonomy and specific threats to selected species. (DOCX) [file pone.0100173.s005.docx]

**Richard K. B. Jenkins et al.: Extinction Risk and Conservation of Madagascar’s Reptiles**

**Supplementary text 1.** Comments on taxonomy and specific threats to selected species

Continued herpetological field work and taxonomic revision in Madagascar will support necessary updates of the extinction risk of reptiles on The IUCN Red List of Threatened Species. Since this analysis was conducted *M. spannringi,* which was known from very few specimens in an area subject to rapid deforestation, has recently been discovered in a littoral forest to the south of its previous range [118]. Furthermore, several new species have been described since 2012 and have not been included in this assessment (Table S1). Some of these are microendemic to very small ranges in north-western or northernmost Madagascar (e.g., *Brookesia confidens, B. desperata, B. micra, B. tristis, Madascincus arenicola, Madagascarophis fuchsi, Sirenoscincus mobydick*), thus probably increasing the number of threatened species overall, and in particular in northern Madagascar [14,119-120]. Other new or resurrected species have large ranges and are unlikely to be threatened e.g. *Furcifer major* and *F. viridis* [121].

Also additional taxonomic changes proposed since completion of the first draft of this paper were not considered but will be considered in updates of the respective species accounts. This regards the transfer of all Malagasy *Typhlops* species to the genus *Madatyphlops*, the transfer of *Ramphotyphlops braminus* to the genus *Indotyphlops*, the inclusion of *Brookesia lolontany* and *B. nasus* in a separate genus *Palleon*, the elevation of *Sanzinia madagascariensis volontany* to full species status, and the synonymization of *Xenotyphlops mocquardi* with *X. grandidieri* that left only a single species in the genus and in the family Xenotyphlopidae. Several Malagasy reptile species have been the subject of more intensive conservation studies or are subjected to very specific threats, warranting additional comments. The ploughshare tortoise *Astrochelys yniphora* is one of the rarest tortoises in the world, restricted to a very small range in western Madagascar [90,94] with an estimated remaining wild population of 600 specimens around the year 2000 [94,102]. A captive breeding and conservation project for this species has been maintained for a considerable period of time in Ampijoroa forest station at Ankarafantsika National Park [93], with some trial reintroductions having been carried out [96], but the wild populations continue to decrease due to the high pressure of the illegal pet trade given the high prices these tortoises can attain. The anticipated extraction of sand deposits threatens the dune-living snake *Xenotyphlops grandidieri*. For other species, industrial mining of ilmenite (*P. antanosy*) and artisanal mining of gold (*Calumma tarzan*) are the primary threats.

The distribution and ecology of many Malagasy reptile species remains poorly understood. The large highland snake *Pseudoxyrhopus ankafinaensis* is known from only a single specimen collected in 1880; it therefore is the only Malagasy reptile recorded in recent times that qualifies as possibly being extinct, especially because its probable original habitat, mid to high-altitude rainforest, has almost completely disappeared at the type locality [115]. A second snake, *Compsophis vinckei,* is known from only two specimens collected in a small area of central eastern Madagascar where slash- and burn agriculture is ongoing, especially outside protected areas. Some species, such as the Critically Endangered snake *Xenotyphlops grandidieri,* and skinks (*Pseudoacontias menamainty, Paracontias fasika, P. minimus*, and *P. rothschildi*) are fossorial and are rarely encountered during fieldwork [116-117].

[for references see main paper].
